# Attitude shifts and knowledge gains: Evaluating men who have sex with men sensitisation training for healthcare workers in the Western Cape, South Africa

**DOI:** 10.4102/sajhivmed.v18i1.673

**Published:** 2017-03-31

**Authors:** Andrew P. Scheibe, Zoe Duby, Ben Brown, Eduard J. Sanders, Linda-Gail Bekker

**Affiliations:** 1 Desmond Tutu HIV Centre, Institute of Infectious Disease and Molecular Medicine, University of Cape Town, South Africa; 2 KEMRI-Wellcome Trust Research Programme, Kilifi, Kenya; 3 Nuffield Department of Medicine, University of Oxford, United Kingdom

## Abstract

**Background:**

Men who have sex with men (MSM) in South Africa experience discrimination from healthcare workers (HCWs), impeding health service access.

**Objectives:**

To evaluate the outcomes of an MSM sensitisation training programme for HCWs implemented in the Western Cape province (South Africa).

**Methods:**

A training programme was developed to equip HCWs with the knowledge, awareness and skills required to provide non-discriminatory, non-judgemental and appropriate services to MSM. Overall, 592 HCWs were trained between February 2010 and May 2012. Trainees completed self-administered pre- and post-training questionnaires assessing changes in knowledge. Two-sample *t*-tests for proportion were used to assess changes in specific answers and the Wilcoxon rank-sum test for overall knowledge scores. Qualitative data came from anonymous post-training evaluation forms completed by all trainees, in combination with four focus group discussions (*n* = 28) conducted six months after their training.

**Results:**

Fourteen per cent of trainees had received previous training to counsel clients around penile–anal intercourse, and 16% had previously received training around sexual health issues affecting MSM. There was a statistically significant improvement in overall knowledge scores (80% – 87%, *p* < 0.0001), specifically around penile–anal intercourse, substance use and depression after the training. Reductions in negative attitudes towards MSM and increased ability for HCWs to provide non-discriminatory care were reported as a result of the training.

**Conclusion:**

MSM sensitisation training for HCWs is an effective intervention to increase awareness on issues pertaining to MSM and how to engage around them, reduce discriminatory attitudes and enable the provision of non-judgemental and appropriate services by HCWs.

## Introduction

The Joint United Nations Programme on HIV and AIDS defines men who have sex with men (MSM) as ‘males who have sex with males, regardless of whether or not they also have sex with women or have a personal or social gay or bisexual identity’.^1^ Although useful, the term MSM classifies males with differing gender expressions, sexual practices and social identities as a single group. This grouping is potentially problematic, as client-centred health interventions are more likely to be effective than interventions that do not take important differences into account. Grouping ‘sub-populations’ of MSM together limits the appropriateness of approaches, for instance, to address differential risks associated with sexual practices (e.g. unprotected penetrative versus receptive penile–anal intercourse) or to overcome stereotypes associated with certain social identities (e.g. homosexual or heterosexual).^2,3,4,5^ In South Africa, MSM have a range of gender expressions, gender identities and sexual practices. Moreover, certain characteristics (including gender expression and sexual practices) may change over time (e.g. from adolescence to adulthood) or by circumstance (e.g. when visiting a family home in a rural area, if in male-only institutions or for financial reasons).^6,7,8^ Yet, gay-identifying males and less ‘visible’ heterosexual and bisexual MSM exist^9,10,11,12,13,14^ in South Africa where constitutional protection conflicts with widespread homophobia.^15,16,17^ Despite the limitations of the term, we employ ‘MSM’ in this article as we present the findings of an evaluation of a sensitisation training programme for healthcare workers (HCWs) in South Africa that focused on MSM.

HIV amongst MSM remains a priority public health concern in South Africa.^18^ HIV prevalence amongst MSM in major metropolitan areas ranges between 13% and 49%^7^; however, no robust MSM population size estimates exist.^11^ Various behavioural, social and structural risk factors contribute to the high HIV burden amongst MSM. Behavioural risk factors include condomless penile–anal intercourse, multiple sex partners and substance use in the context of sexual encounters.^6,11,14,19,20,21,22^ Social and structural risk factors include stigma and discrimination towards MSM,^23^ low levels of education and high rates of poverty amongst MSM from previously disadvantaged communities^6^ and limited access to MSM-appropriate health services.^23^ Discrimination and internalised homophobia are correlated with increased risk-taking behaviour amongst MSM.^24,25^ Further, MSM often experience discrimination from public sector HCWs,^23,26^ negatively affecting health-seeking behaviours and disclosure of risk practices.^27^

For the purposes of this article, we use the word ‘sensitisation’ to refer to the process of increasing awareness and knowledge of an issue to instil empathy and effect the modification of negative attitudes and behaviour, with the intention of reducing discrimination and inequality. Sensitisation training assists people in examining their personal attitudes and beliefs.^28^ Sensitisation training for HCWs around MSM is recommended by the World Health Organization^29^ and has been employed by other organisations in South Africa and in African other countries (e.g. Nigeria and Malawi)^30^; however, we were only able to find published data on the evaluation of the HCW training from Kenya (discussed later),^31^ which was found to increase knowledge and support empathetic healthcare responses amongst HCWs towards MSM.^31^

## The training intervention

In 2009, the Desmond Tutu HIV Foundation (Cape Town, South Africa) and the KEMRI-Wellcome Trust Research Programme (Kilifi, Kenya) developed a training manual to equip HCWs with the knowledge, awareness and skills to provide sensitive and appropriate services to MSM.^32^ HCWs were conceptualised to include clinical (e.g. nurses, doctors and counsellors) and support staff (e.g. clinic managers, outreach workers and social workers). Counsellors and nurses were the primary audience for the training.

In 2011, the manual was revised based on recommendations from previous training participants and included updates around HIV epidemiology, advances in biomedical prevention research and additional case studies and interactive exercises.^33^ The training manual covered homophobia, stigma and discrimination, HIV and sexually transmitted infections (STIs), sexual orientation and gender identity (including a detailed explanation of the term MSM), common sexual practices, condoms and lubricants, mental health issues, substance use and risk-reduction counselling for MSM. The training did not include capacity development around the clinical management of health and related conditions affecting MSM.

We used the manual to provide sensitisation training to 592 public- and private-sector HCWs in the Western Cape province of South Africa, between February 2010 and May 2012. Training sessions were conducted over one or two days, depending on operational requirements, and included 10–20 trainees per training. The training programme was advertised by means of electronic communication and word of mouth. Training workshops were conducted in English and combined facilitator-led presentations, interactive group exercises, question and answer sessions, and opportunities for individual self-reflection. Training was provided free of charge and each trainee received a manual.

## Research design

Quantitative and qualitative methods were used to evaluate the training programme.

### Quantitative component

Trainees completed written pre- and post-training assessment questionnaires, before the start of, and at the end of, training. Pre-training assessments included previous training around MSM sensitisation, penile–anal intercourse and counselling MSM clients. Pre- and post-training assessments were taken from the MSM training manual and examined the sensitivity training manual curriculum.^34^ In short, the trainees’ baseline knowledge relates to common STIs amongst MSM; stigma, discrimination and homophobia and their effects; common sexual practices amongst MSM; common mental health problems experienced by MSM; and condom and lubricant use for penile–anal intercourse. Post-training assessments examined trainees’ perceptions of the need for HCWs to be sensitive to MSM-related issues, MSM-appropriate service provision, personal beliefs and attitudes as well as comfort in counselling MSM around penile–anal intercourse. The assessments included closed questions with categorical variables (true or false; yes or maybe or no). A score was calculated based on the 15 knowledge questions included in the training manual. Trainees’ demographic data were not collected, and participants were not requested to write down any identifying information on their assessment forms. Data from the assessment forms were entered into an Excel spread sheet and imported into Stata Corp v 11.2 (College Station, Texas). Pre- and post-training proportions for correctly answered knowledge-related questions were calculated and compared using the two-sample *t*-test for proportions. Changes in average pre–post training knowledge scores were assessed using the Wilcoxon rank-sum test. It was not possible to link pre- and post-assessments to individuals as participant details were not captured.

### Qualitative component

All trainees completed a semi-structured, anonymous written evaluation form immediately after the training. The data from these forms were typed up verbatim and included in the qualitative analysis, alongside the data from focus group discussions (FGDs). Attempts were made to contact all of the HCWs who attended training in Cape Town between February and May 2010 (*n* = 167) via email or telephone. Trainees were then invited to take part in an FGD six months after receiving the training. Twenty-eight of the trainees (17%, 28/167) took part, and FGDs were scheduled to suit their availability. Four semi-structured FGDs were held in Cape Town during October and November 2010. FGD respondents included 18 counsellors, two nurses, four management-level HCWs, an outreach worker, one social worker and two people who did not stipulate their position. Topics of discussion included self-perceived attitude shifts towards MSM, experiences since the training in providing services to MSM clients, and providing services relating to penile–anal intercourse. The lead trainer conducted the FGDs in English. FGDs were audio recorded and accompanied by written notes. Audio recordings were transcribed verbatim, with identifying information omitted. An iterative open-coding analysis process was used, which involved comparing, contrasting and conceptualising the data across key emergent themes. One person conducted all of the coding. Qualitative analysis followed a grounded theory approach based upon the generation of provisional hypotheses and theories from the data, with subsequent elaboration and verification stages. ‘Manifest’ meaning was obtained through analysis of the key themes and ‘latent’ meaning from reflection on these and the findings from other components of the evaluation process, the context, and after discussion between the primary and secondary authors.

#### Ethical consideration

Approval for this evaluation was granted by the University of Cape Town’s Faculty of Health Sciences Human Research Ethics Committee. Written informed consent to use the information collected was obtained from trainees. Personal details of the trainees were not captured on evaluation forms or procedures to protect their identity.

## Findings

### Quantitative component

A total of 592 HCWs participated in the MSM sensitisation training between February 2010 and May 2012. A total of 495 pre-training and 468 post-training assessments were completed and included in the analysis. Twelve trainees did not complete all of the questions.

Few trainees had received previous training on how to counsel clients around penile–anal intercourse or on sexual health issues affecting MSM (14%, 69/495 and 16%, 75/495, respectively). Fewer than half (42%, 200/495) had ever asked a male client about sex with another man.

Overall, there was a statistically significant increase in the composite knowledge scores, increasing from a median score of 80% at pre-training to 87% at post-training (*p* < 0.0001). An overview of pre–post training scores per assessment question is provided in Table 1. There was a statistically significant increase (*p* < 0.05) in the number of trainees who correctly answered the questions around stigma, HIV and MSM, anal sex practices amongst non-MSM, the relative risk of condomless anal sex, awareness of STIs affecting MSM, substance use and depression after the training. After the training, 75% of the trainees reported that they felt comfortable asking future clients about penile–anal intercourse, and 96% reported that they were aware of the potential risks associated with common sexual activities that MSM may engage in.

**TABLE 1:** Comparison of pre–post training knowledge scores.

Item		Pre-training		Post-training		Two-sample *t*-test of proportion (*p*-value)
	
		%	Score	%	Score	
1.	Acknowledge that STIs, including HIV, are a major problem amongst African men	85	397/466	84	372/443	0.4329
2.	Acknowledge that research in Africa shows that sex between men does occur	92	433/472	91	415/455	1.0000
3.	Acknowledge that many countries in Africa have laws that criminalise homosexuality	94	436/465	92	424/460	0.2831
4.	Acknowledge that stigma against MSM places them at risk for acquiring HIV	85	391/462	95	431/454	<0.0001
5.	Understand that *coming out* means letting others know about a person’s sexual orientation or practices	89	417/469	91	417/459	0.0588
6.	Acknowledge that anal sex is not only practiced by MSM	78	367/473	90	414/461	<0.0001
7.	Acknowledge that unprotected (condomless), receptive anal intercourse carries highest risk of getting HIV	92	432/469	96	441/461	0.007
8.	Acknowledge that HIV, herpes, gonorrhoea and chlamydia are STIs which affect MSM	84	390/466	89	412/463	0.0006
9.	Acknowledge that excess feelings of fear can cause mental health problems, which are common amongst MSM	74	339/459	91	420/460	<0.0001
10.	Acknowledge that symptoms of depression can include sadness, feeling down and changes in sleeping and eating patterns	94	445/473	98	455/462	<0.0001
11.	Acknowledge that alcohol and drug use is common amongst MSM	74	343/467	93	425/458	<0.0001
12.	Acknowledge that alcohol and drug use has been associated with increased risk of HIV	94	448/475	97	450/462	0.0049
13.	Acknowledge that MSM with harmful patterns of substance use should be referred to a professional for further management if possible	90	420/468	97	446/462	<0.0001
14.	Acknowledge that if used correctly, condoms can prevent most STIs	98	467/478	98	455/462	0.3257
15.	Acknowledge that oil-based lubricant should not be used with latex condoms	81	376/467	84	385/461	0.2251
**Overall knowledge score (%)**		**80**	**-**	**87**	**-**	**<0.0001**

MSM, men who have sex with men; STI, sexually transmitted infection.

### Qualitative component

The following section presents qualitative data collected from the post-training evaluation forms and the four FGDs. The qualitative data fall into four thematic areas: (1) reflections and perceptions of changes in knowledge and shifts in attitude, (2) implications of these on service provision and care, (3) barriers to service provision and (4) recommendations for implementation.

#### Effect of the training on knowledge and attitudes

Religious and cultural beliefs were cited by many respondents as sources of their own prejudice about homosexuality being sinful. The general view expressed was that the training resulted in a positive shift towards increased sensitivity and acceptance of MSM and the diversity of human sexuality:

‘The course was very informative, alerting people to know information which helps in breeding tolerance and understanding amongst people.’ [Anonymous post-training evaluation form]

Respondents reported a ‘change of mind-set’ towards MSM and used terms such as ‘eye opening’ and ‘mind opening’ to describe the training:

‘After the training, I was more open minded about these things and more comfortable… I remember I was watching TV, 2 or 3 years back… there was this movie about these gay people who were having sex… and I was like [makes shocked gasping noise], I nearly vomited, and I had like this funny feeling in the bottom of my stomach, I was like ‘dear god, what is going on, what are there men doing?’ …I have had a change of attitude towards it now.’ [Female, FGD]

Also reported were increases in MSM-related knowledge and awareness of the social discrimination and exclusion experienced by MSM:

‘Now I really feel equipped on how to deal with MSM because I was really in the dark, so I’m grateful for this knowledge I gained here.’ [Anonymous post-training evaluation form]

Respondents described their increased sense of compassion for MSM and the social stigmatisation, discrimination and exclusion in the community setting, rejection from family and friends and the resulting loneliness, depression and feelings of isolation and hopelessness experienced by many MSM:

‘I’d never really thought about how they [MSM] feel and all the other things. After doing the training, I had more understanding of how they feel… It helped change my attitude… and to really think about the pain they go through as individuals.’ [Anonymous post-training evaluation form]

Some respondents said that the training made them reflect on social norms and the heteronormativity of society:

‘[The training was] really insightful. It helped me open my mind and broaden my understanding of MSM and the social norms we as society enforce on people.’ [Anonymous post-training evaluation form]

Some knowledge gains and shifts in attitude went beyond gay men and other MSM, having broader relevance to other non-heterosexual sexualities including lesbians and other women who have sex with women:

‘I will be at ease when a couple will come to me and talk about their different sex ways, because of today.’ [Anonymous post-training evaluation form]

Not all of the FGD participants felt that their attitudes had changed dramatically as a result of the training:

‘I don’t think it was a major shift but it has made me more comfortable.’ [Female, FGD]

A few HCWs commented that initially, they had found some of the training content challenging, shocking and in conflict with their personal views and beliefs:

‘I am a bit shocked but the training was very informative.’ [Anonymous post-training evaluation form]

Many respondents felt that the training content and activities had been conducive to learning, sharing experiences and exploring personal attitudes and beliefs, and how these relate to service provision:

‘The activities made it easier to talk about the topic. It highlighted how personal views can influence how one provides services to MSM. I liked the fact that the workshop did not try and change my perceptions but focused on how MSM need help and assistance.’ [Anonymous post-training evaluation form]

#### Implications for service provision and care

An increased sense of compassion and understanding was attributed to the training, along with a desire to implement changes in their work place to provide non-judgemental services:

‘I’m not one of them but I’m comfortable talking about it. I want to protect my clients, and make them safe… We can’t tell people it’s wrong, we can’t judge them, we’re not judges, we’re carers. So I just do my work.’ [Female, FGD]

Respondents admitted to their previously held beliefs that only gay men have anal sex; many said that they had never thought to ask either ‘straight’ male or female clients about penile–anal intercourse previous to the training:

‘I’ve never asked them [clients] about anal sex before. After the training it really broadens [you] up and you realise you do need to ask because we do have… [straight/heterosexual] couples who do prefer to have anal sex, and they are comfortable with that, so we need to respect them and actually advise them as to the risk factors, and show them how to make it safe.’ [Female, FGD]

The perception of feeling more comfortable in discussing penile–anal intercourse and same-sex practices after receiving the training was a commonly felt attitude shift amongst trainees. Respondents used phrases like ‘more at ease’, ‘more comfortable’ and ‘more empowered’ to describe their feelings towards having MSM clients and talking about anal sex generally after the training:

‘The training has helped me so much… it has changed me a lot. Mostly around anal sex, I didn’t know about it before. At least now I can explain, I can tell, because of the trainings I received. So I’m comfortable now to talk about it.’ [Male, FGD]

Trainees who work directly with clients in the healthcare setting remarked on their realisation that they had been making heterosexist assumptions about their client’s sexual partner or partners or practices without asking the client themselves:

‘It’s not only with the MSM clients, it’s with the clients who come there in general. That you go into the room not just assuming that… because of their gender, they’re going to choose the opposite sex… so you go in there with that in the back of your mind. So the kinds of questions you ask now is a little different than before. Because before you were thinking, ok so this is a woman client, so you’re always asking her about her husband or boyfriend. So instead of saying that I now, ask about ‘the partner’, because the partner can be whoever [either gender]… it [the training] does broaden your way of asking things and approaching the client.’ [Female, FGD]

Many of the respondents described their new-found acceptance of the diversity of sexual preferences and practices, and the importance of endeavouring not to judge, stigmatise or discriminate against clients:

‘Before I would have goose bumps [hearing about two men having sex]… ‘Man, how does this happen? Ugh’ – before I tended to have that attitude, because I didn’t really understand and know why would a person choose this [having sex with someone of same-sex]. For me, that was not normal… But now it’s so normal, like ‘ok, this is what the person chooses to do’… I’ve come to realise that behind closed doors you can explore, you can do whatever, it doesn’t matter whether you’re MSM or heterosexual, you choose to do what you do, and I accept the person’s choice.’ [Female, FGD]

Those respondents who felt that they had been relatively well informed and non-judgemental before the training said that their new knowledge gains served to increase their motivation to provide MSM-sensitive services:

‘It’s not necessarily that I’ve changed, but the training motivated me more… having that manual and applying that and the knowledge, I have become more motivated.’ [Male, FGD]

There was recognition from some respondents of the need for the training even though the South African Constitution protects the rights of all people regardless of sexuality or gender:

‘This training is very important and covers almost every aspect on working with MSM. This is really needed as our country is now sensitive and acknowledges MSM, so as means of our long-fighting battle of reducing the growing number of HIV, this MSM training is needed.’ [Anonymous post-training evaluation form]

#### Experiences providing services to men who have sex with men after the training

A general feeling amongst FGD respondents was that since the training, they had been able to provide better services to MSM clients, as a result of being more informed, equipped with appropriate skills and knowledge, and prepared to ask appropriate questions of their clients:

‘I think it’s almost a relief for a lot of people to actually be able to tell someone who’s not going to pass judgement. They go off and tell you just about everything you want to know… Once you ask the questions the person is comfortable and then they come out… the person is relaxed now.’ [Female, FGD]

Respondents reported that they also felt more comfortable in the company of MSM clients and discussing penile–anal intercourse; as a consequence, clients felt more comfortable to disclose their sexual behaviour:

‘I’ve had two MSM couples, and they were free and talking, they felt the environment was free, they were talking about everything, so I’m so glad also that the couples are also coming in, and the MSM can feel free to express everything.’ [Female, FGD]

Some of the HCWs reported that after receiving the training, they had initially been concerned about having to ask clients about anal sex:

‘I thought when we initially asked the anal sex question that people would be affronted, but surprisingly not… those who don’t [have anal sex] will just say ‘no’ [sounding surprised] and then you just move on. I was quite surprised… I do warn people that it’s quite personal the questions, and they have to see it in a broad context, because we have to speak to everybody, and this is just to see people’s sexual behaviour and so on… they seem to sort of take to it OK.’ [Female, FGD]

#### Barriers to service provision

In terms of barriers to service provision for MSM, one respondent stated that even if HCWs are sensitised about anal sex, the risk assessment forms used in the public sector do not include questions on anal sex, and therefore, the HCWs will not ask about it:

‘We’ve had people saying to us that they won’t ask the questions if they are not on the forms, because they don’t know how to ask them.’ [Female, FGD]

Overcoming HCWs’ personal beliefs and opinions was identified as important to provide necessary services to clients:

‘I am an individual, and I have my values and principles, in terms of that, I do find a challenge here and there, but I’ve learned that these are my values, and this is the respondent, so I mustn’t push my values on them, so let’s focus on them and help them to be where they’re at without being judgemental.’ [Male, FGD]

‘You don’t expect it from healthcare workers… they need to learn to put their personal issues aside and be professional… Who are we there for? We are there for our clients. It’s so strange to work with people who still have that attitude.’ [Female, FGD]

### Recommendations for implementation

A commonly occurring recommendation was that the existing tools used for risk assessment and screening by clinics needed to be revised to include questions on penile–anal intercourse, anal STIs and MSM behaviour.

Another commonly cited issue was the lack of informative and educational materials relating to MSM, sexuality and penile–anal intercourse available in health facilities. Respondents felt that the visibility of such materials would make clients feel comfortable and would encourage disclosure:

‘People will sit there and read (posters and pamphlets)… it will encourage them to talk about it. But if they don’t see it anywhere, they won’t now come and ask the sister.’ [Female, FGD]

In terms of training requirements, respondents suggested that follow-up refresher trainings would be useful, combined with online reference and referral resources. It was also suggested that sensitisation of all levels of staff working in health facilities needs to take place:

‘We need to bring all levels of staff in order for the changes to happen. Even starting from the security, if the security is discriminating, they (MSM) won’t even come inside. The receptionist, everyone who works inside, the doctors and nurses too.’ [Male, FGD]

## Discussion

The findings of this research suggest that a two-day or less, low-cost, in-service sensitisation training for HCWs can reduce homo-prejudicial attitudes and increase relevant knowledge and relational skills. This training intervention increased knowledge and awareness of the health risks, social vulnerabilities and specific needs of MSM amongst those who completed assessment forms. However, the quantitative elements of the assessment forms did not assess shifts in values or moral views. Increased HCW skills and comfort in providing services to MSM was identified through the qualitative methods. Focus group participants reported shifts towards being less judgemental and discriminatory towards MSM in their healthcare settings. It is likely that changes such as these will help to reduce barriers impeding MSM from accessing health services.

The relatively low number of trainees that had counselled MSM prior to receiving the training may indicate that MSM had not been accessing HIV services at that time. Another potential explanation is that MSM who were accessing health services may not have disclosed same-sex behaviours to HCWs. Furthermore, very few trainees had received previous training around penile–anal intercourse, despite penile–anal intercourse being the most efficient way of transmitting HIV sexually.^35^

The baseline knowledge of participants appeared to be higher than the baseline knowledge amongst HCWs in Kenya who were trained using the same curriculum.^24,31^ Constitutional protection against discrimination on the basis of sexual orientation and gender^36^; government policy around non-discriminatory public service provision (*Batho Pele Principles*)^37^; inclusion of MSM in the National Strategic Plan for HIV, STI and TB^38^; and existence of an established lesbian, gay, bisexual and transgender ‘community’ within the Western Cape’s major metropolitan area (Cape Town)^9^ may have contributed to the higher levels of knowledge amongst HCWs in the Western Cape compared with the Kenyan counterparts.

Healthcare workers admitted to previously making heterosexist assumptions when conducting sexual risk assessments, an issue that has been previously identified as limiting disclosure of same-sex sexual behaviour.^39^ General knowledge around the social, structural and health vulnerabilities of MSM was also low, highlighting the need for improvements in HCW knowledge around MSM-specific sexual health information. The training and evaluation exposed judgemental, moralising and homo-prejudicial attitudes amongst some HCWs towards MSM, demonstrating the challenges involved in creating an enabling environment for MSM to access healthcare, as well as the limitations of once-off sensitisation training interventions. The qualitative evaluation revealed some shifts in attitudes; however, on several occasions, HCWs referred to MSM in the third person, suggesting that much work is needed for sexual minority groups to be embraced by them.

Religious and cultural beliefs play a key role in informing people’s attitudes towards homosexuality.^40,41^ It is imperative that healthcare providers distinguish between their right to hold personal values and beliefs, and their professional obligation to render services free of prejudice and discrimination; it is a requirement of professionalism that personal beliefs be set aside when they conflict with professional duty.^40,42^ One element of sensitisation training involves making HCWs aware of the distinction between their right to ‘hold personal values, beliefs and prejudices’, with ‘their professional obligation to render services free of prejudice and/or discrimination’.^42^

The evaluation of the MSM sensitisation training programme for HCWs in Kenya found similar results regarding the effects of training.^24^ The two-day training intervention in Kenya, which included computer-based sessions followed by group discussions, significantly increased trainee knowledge on issues relating to MSM.^24^ Notable knowledge gains related to the following areas: the effects that stigma and discrimination towards MSM can have on their vulnerability to HIV; penile–anal intercourse is practiced by men and women regardless of their sexuality, rather than only between MSM; the physiology of penile–anal intercourse, and related STI and HIV transmission risks; and increased awareness around mental and sexual health issues affecting MSM.^24^

The evaluation highlighted how training alone is unlikely to shift practices and that it needs to be accompanied by institutional changes, including provision of tools that HCWs can use to implement the new knowledge (e.g. sexual reproductive health assessment forms that are gender neutral and can capture a range of identities, genders and sexual practices). Sensitisation training is an important step towards appropriate health services for MSM. In addition, skills to appropriately manage health conditions of particular relevance for MSM need to be developed and has started to take place in South Africa.^30,43,44^

### Limitations of the study

Limitations to this research included the following: (1) we were unable to link pre- and post-assessment questionnaires to individual trainees, and as a consequence, limited reflections can be made on changes in individual trainees versus the group; (2) trainee demographic information was not recorded on the assessments, preventing further analysis; (3) response bias and the willingness of trainees to frankly discuss the programme and provide criticism without concern for causing offence are likely as the lead trainer also facilitated the FGDs; (4) FGD respondents were trainees who agreed to return after the training, which may have led to sampling bias – the trainees who agreed to participate in the qualitative aspects of the evaluation may have been more positive about the training; and (5) the evaluation did not assess the long-term effects of the training, and it is not possible to say if changes identified in the evaluation were durable.

Another potential limitation, unrelated to the research methods, but to the training itself, was that the facilitator was an English-speaking (not isiXhosa or Afrikaans, the two predominant first languages in the Western Cape) woman. Notably, the trainings were not facilitated by an openly gay man, as such, the female facilitator may not have been able to accurately relate the concerns or priority issues of MSM. Arguably, the positioning of the trainer as a neutral ‘outsider’ may have made it easier for trainees to be open and honest about their prejudices.^45^

The term ‘MSM’ groups men together with different sexual practices and risk, social identities and gender expressions. Although it is useful, the term and approach have limitations. Future training programmes and subsequent service delivery would be improved if greater attention were paid to the various sub-groups that the MSM term encompasses, and how sexual practices, gender identity and sexual orientation are influenced by a range of factors, which may also change over time.

## Conclusion

The findings of this study evaluating the MSM sensitisation training for HCWs in the Western Cape province of South Africa suggest that this training intervention increased knowledge around MSM issues, reduced judgemental and homo-prejudicial attitudes, and improved self-perceived skills and capacity in providing appropriate and relevant healthcare to MSM. The training was particularly beneficial in increasing awareness of MSM and knowledge relating to the issues of importance for MSM, specifically HIV, STIs, social stigma, mental health, substance use, sexual risk behaviours, anal health, and correct condom and lubricant use.

These findings suggest that this type of sensitisation training should be scaled up nationally and integrated into health service provider training. MSM sensitisation training for HCWs is an effective intervention to enable the provision of non-judgemental and appropriate services by HCWs and to increase awareness of unique issues pertaining to MSM and how to manage them. However, as seen from these results, even if HCWs are sensitised, without improved reporting systems and tools that are more inclusive and disaggregated, questions relating to sexuality and anal sex will not be asked, thus hampering effective service provision.

Focus should remain on ensuring that training and reform, which support the implementation of training content, contribute to enhanced access to quality care for MSM. Efforts to increase the efficiency of sensitisation training and to measure the long-term effectiveness thereof are also needed. Training should include other stigmatised groups at risk for HIV and should include additional focus on clinical aspects, including the management of anal STIs amongst patients. To be sustainable, training around MSM and related issues should be included in HCW pre-service training.
